# Supplementary material for: Prognostic factors of first intimate partner violence among ever-married women in Sub-Saharan Africa: Gompertz gamma shared frailty modeling
Source: PLoS One. 2024 May 31;19(5):e0303187. doi: 10.1371/journal.pone.0303187 (PMC11142580; doi:10.1371/journal.pone.0303187)
Supplement: Attachment — Submitted filename: Point by point response.docx [file pone.0303187.s001.docx]

**Point-by-point response**

**Point by point response for editors/reviewers comments**

PLOS ONE

**Manuscript title:**  Prognostic factors of first intimate partner violence among ever-married women in Sub-Saharan Africa: Gompertz gamma shared frailty modeling

**Manuscript ID:**   PONE-D-23-11923

**Dear editor/reviewer.**

Dear all,

We would like to thank you for the constructive, building, and improvable comments on this manuscript that would improve the content of the manuscript. We considered each comment and clarification question of editors and reviewers on the manuscript thoroughly. Our point-by-point responses for each comment and question are described in detail on the following pages. Further, the details of changes were shown by track changes in the supplementary document attached

**'Response to Reviewers**

**Editor Comments:**

1. Please ensure that your manuscript meets PLOS ONE's style requirements, including those for file naming. The PLOS ONE style templates can be found at

<https://journals.plos.org/plosone/s/file?id=wjVg/PLOSOne_formatting_sample_main_body.pdf> and <https://journals.plos.org/plosone/s/file?id=ba62/PLOSOne_formatting_sample_title_authors_affiliations.pdf>

**Author’s response:** Dear editor thank you for your comment. We have ensured that the manuscript is written according to PLOS ONE's style requirements, including those for file naming.

2. Please provide additional details regarding participant consent. In the ethics statement in the Methods and online submission information, please ensure that you have specified (1) whether consent was informed and (2) what type you obtained (for instance, written or verbal, and if verbal, how it was documented and witnessed). If your study included minors, state whether you obtained consent from parents or guardians. If the need for consent was waived by the ethics committee, please include this information.

If you are reporting a retrospective study of medical records or archived samples, please ensure that you have discussed whether all data were fully anonymized before you accessed them and/or whether the IRB or ethics committee waived the requirement for informed consent. If patients provided informed written consent to have data from their medical records used in research, please include this information

Once you have amended this/these statement(s) in the Methods section of the manuscript, please add the same text to the “Ethics Statement” field of the submission form (via “Edit Submission”).

For additional information about PLOS ONE ethical requirements for human subjects research, please refer to <http://journals.plos.org/plosone/s/submission-guidelines#loc-human-subjects-research>.

**Author’s response:** Dear editor thank you for your comment. Data access permission was received from the measure DHS program via an online request at <http://www.dhsprogram.com> . The data used in this research were publicly available and did not contain any personal identifiers. We have acknowledged this in the “ethics statement” of the method section of the manuscript. (please see the revised manuscript).

3. We note that you have stated that you will provide repository information for your data at acceptance. Should your manuscript be accepted for publication, we will hold it until you provide the relevant accession numbers or DOIs necessary to access your data. If you wish to make changes to your Data Availability statement, please describe these changes in your cover letter and we will update your Data Availability statement to reflect the information you provide.

**Author’s response:** Thank you dear editor for your suggestion. The dataset used in this study is available online and can be accessed at [www.measuredhs.com](http://www.measuredhs.com).

4. Please review your reference list to ensure that it is complete and correct. If you have cited papers that have been retracted, please include the rationale for doing so in the manuscript text, or remove these references and replace them with relevant current references. Any changes to the reference list should be mentioned in the rebuttal letter that accompanies your revised manuscript. If you need to cite a retracted article, indicate the article’s retracted status in the References list and also include a citation and full reference for the retraction notice.

**Author’s response:** Thank you dear editor for your suggestion. We have verified that all the references cited in this study remain valid and have not been retracted.

**Response to reviewer 1**

Reviewer #1: The manuscript is technically sound and uses fancy statistics but does not answer the research question, namely 'Therefore, this study aimed to investigate the timing of first intimate partner violence (FIPV) among ever-married women in 30 SSA countries and to identify the risk factors of the timing by using the appropriate statistical analysis method.'

**Author’s response:** Dear reviewer thank you for your comment. We have answered our research question to ‘investigate the timing of first intimate partner violence (FIPV) among ever-married women in 30 SSA countries and to identify the risk factors of the timing by using the appropriate statistical analysis method.' Indeed, survival analysis is a powerful tool for addressing research questions that involve time-to-event outcomes. By considering the time component and estimating hazard ratios, we gain insights into the factors influencing the outcome, timing of the first intimate partner violence. Furthermore, we calculated the person-time-at-risk, which can provide us the information the rate of the FIPV or how quickly women are experiencing the FIPV following their marriage.

Methods: The present study has utilized 125,731 weighted samples, BUT it does not say from which countries, which is not helpful for targeted recommendations because countries differ.

**Author’s response:** Dear reviewer than you for your comment. We have included a table representing the list of countries with their respective weighted frequency and proportion of intimate partner violence across the included countries.

Results. Nothing new. The problem has been rehashed many times. These statistics are not unknown. Perhaps if we understood better when in the marriage the violence was initiated and the circumstances, interventions could be better targeted. Thus the authors need to present 'the timing of first intimate partner violence (FIPV) among ever-married women' and 'risk factors of the timing ' AND whether this is similar or how it differs in the 30 countries.

**Author’s response:** Dear reviewer thank you for your comments. Prior research has focused on intimate partner violence within individual SSA nations, examining significant predictors. In our current study, we aim to provide a broader SSA-level perspective by investigating the incidence and predictors of first intimate partner violence among all married women. Our use of a representative sample size ensures robust statistical power and relatively unbiased estimates. These findings hold valuable implications for policymaking and targeted interventions across SSA nations. When dealing with heterogeneity, we often use random effects models (frailty in our case). These models incorporate random effects (such as country-specific intercepts) to account for variability between different units (e.g., countries).

The estimated median survival remains elusive, as more than half of the individuals have yet to encounter their first instance of intimate partner violence (FIPV). In this context, calculating a median survival is not feasible. However, we can get insights from the overall incident rate and the incidence rate of FIPV over 5-year intervals within marriages or unions. These rates provide valuable information about the occurrence and patterns of FIPV across different durations of relationships.

EXAMPLES OF ENGLISH CORRECTIONS REQUIRED.

Line 177. majority (64.95%) of them were from rural residence,

Line 178,, More than half (69.56%) of the women has a decision making autonomy

**Author’s response:** Thank you dear reviewer. We have corrected.

**Response to reviewer 2**

Arrange the "Key word" alphabetically (line 50)

**Author’s response:** Dear reviewer thank you for your comment. We have arranged in ascending alphabetical order.

The introduction was well written highlighting the issue and the gaps of previous studies that necessitate this study. The objective of the study is clear.

**Author’s response:** Thank you for your kind words! We appreciate your compliment.

Method

The design of the study is clearly stated--DHS & cross-sectional. The outcome and independent variables was clearly spelt out and the methodology is well written

**Author’s response:** Thank you for your kind words! We appreciate your compliment.

Discussion

was the statement in line 228-229 a result from this study? If yes, please recast to capture so.

**Author’s response:** Thank you dear reviewer for your concern. Yes, the statement was based on our finding and we have recast it.
