# Supplementary material for: Prognostic factors of first intimate partner violence among ever-married women in Sub-Saharan Africa: Gompertz gamma shared frailty modeling
Source: PLoS One. 2024 May 31;19(5):e0303187. doi: 10.1371/journal.pone.0303187 (PMC11142580; doi:10.1371/journal.pone.0303187)
Supplement: Attachment — Submitted filename: Response to Reviewers.docx [file pone.0303187.s002.docx]

**Point-by-point response**

**Point by point response for editors/reviewers comments**

PLOS ONE

**Manuscript title:**  Prognostic factors of first intimate partner violence among ever-married women in Sub-Saharan Africa: Gompertz gamma shared frailty modeling

**Manuscript ID:**   PONE-D-23-11923R1

**Dear editor/reviewer.**

Dear all,

We would like to thank you for the constructive, building, and improvable comments on this manuscript that would improve the content of the manuscript. We considered each comment and clarification question of editors and reviewers on the manuscript thoroughly. Our point-by-point responses for each comment and question are described in detail on the following pages. Further, the details of changes were shown by track changes in the supplementary document attached

**'Response to Editor**

**Editor Comments:**

1. Additional Editor Comments:

The paper presents an important investigation into the timing of first intimate partner violence (FIPV) among ever-married women in 30 Sub-Saharan African countries. The utilization of the Gompertz gamma shared frailty model to identify predictors of FIPV is commendable and contributes to the understanding of intimate partner violence (IPV) in low and middle-income countries.

Although the reviewers have accepted the paper, I have couple of concerns below:

The inclusion of variables with p-values less than 0.2 in the bivariable analysis deviates from the standard practice, where only significant variables (p < 0.05) are typically forwarded to the multivariate analysis. I recommend that the authors reanalyze the data, considering only significant variables (p < 0.05) for inclusion in the multivariable analysis.

**Author’s response:** Dear Academic editor thank you for your comment. We have made some changes to the method section based on the results of our analysis. Although we previously stated that we included variables with a p-value less than 0.2, we have now removed this statement as all variables in the bivariable analysis were statistically significant (p<0.05) with a 95% confidence interval, as shown in Table 5. We have reported the Crude Hazard Ratio (CHR) along with its respective 95% CI.

I am interested to know the clarification on why Gompertz gamma shared frailty modelling was chosen over proportional hazard modelling. Providing justification for this choice in the manuscript would enhance the readers' understanding of the methodology employed in the study.

**Author’s response:** Frailty models are used to account for unobserved heterogeneity or unmeasured factors that affect the hazard rate in survival analysis. These models introduce a random effect (frailty) that captures individual-specific variations not explained by observed covariates. Shared frailty models assume that the frailties are correlated across individuals within a group (clusters in our case). When the **global Schoenfeld residuals test** yields a **p-value less than 0.05**, it indicates that the **proportional hazards (PH) assumption** has been violated. Consequently, relying solely on a proportional hazard model (PHM) would not be appropriate in this scenario. The violation of the PH assumption implies that the hazard ratios for the covariates are not constant over time. In such cases, alternative survival models that account for time-varying effects or unobserved heterogeneity become necessary. The **Gompertz gamma shared frailty model**, which incorporates random frailties to capture individual-specific variations, were a suitable choice.
